# An assessment of adaptation and fidelity in the implementation of an audit and feedback-based intervention to improve transition to adult type 1 diabetes care in Ontario, Canada

**DOI:** 10.1186/s43058-024-00563-2

**Published:** 2024-03-18

**Authors:** Syed Zain Ahmad, Noah Ivers, Ian Zenlea, Janet A. Parsons, Baiju R. Shah, Geetha Mukerji, Zubin Punthakee, Rayzel Shulman

**Affiliations:** 1 Institute of Medical Science, Temerty Faculty of Medicine, University of Toronto, Toronto, Canada; 2 SickKids Research Institute, Toronto, Canada; 3 Women’s College Institute for Health System Solutions and Virtual Care, Toronto, Canada; 4 Department of Family Medicine, Women’s College Hospital, University of Toronto, Toronto, Canada; 5 Institute for Better Health, Trillium Health Partners, Mississauga, Canada; 6 Department of Occupational Science & Occupational Therapy, Temerty Faculty of Medicine, University of Toronto, Toronto, Canada; 7 Li Ka Shing Knowledge Institute, St. Michael’s Hospital, Unity Health Toronto, Toronto, Canada; 8 Department of Medicine, Temerty Faculty of Medicine, University of Toronto, Toronto, Canada; 9 Division of Endocrinology, Sunnybrook Health Sciences Centre, Toronto, Canada; 10 ICES, Toronto, Canada; 11 Women’s College Institute for Health Systems Solutions and Virtual Care, Women’s College Hospital, Toronto, Canada; 12 Department of Medicine, McMaster University, Hamilton, Canada; 13 Division of Endocrinology, The Hospital for Sick Children, Toronto, Canada; 14 Department of Pediatrics, University of Toronto, Toronto, Canada

**Keywords:** Quality improvement, Transitions of care, Implementation science, Pediatric diabetes, Adaptation, Fidelity, Community of practice

## Abstract

**Background:**

The fit between an intervention and its local context may affect its implementation and effectiveness. Researchers have stated that both fidelity (the degree to which an intervention is delivered, enacted, and received as intended) and adaptation to the local context are necessary for high-quality implementation. This study describes the implementation of an audit and feedback (AF)-based intervention to improve transition to type 1 diabetes adult care, at five sites, in terms of adaptation and fidelity.

**Methods:**

An audit and feedback (AF)-based intervention for healthcare teams to improve transition to adult care for patients with type 1 diabetes was studied at five pediatric sites. The Framework for Reporting Adaptations and Modifications to Evidence-based Implementation Strategies (FRAME-IS) was used to document the adaptations made during the study. Fidelity was determined on three different levels: delivery, enactment, and receipt.

**Results:**

Fidelity of delivery, receipt, and enactment were preserved during the implementation of the intervention. Of the five sites, three changed their chosen quality improvement initiative, however, within the parameters of the study protocol; therefore, fidelity was preserved while still enabling participants to adapt accordingly.

**Conclusions:**

We describe implementing a multi-center AF-based intervention across five sites in Ontario to improve the transition from pediatric to adult diabetes care for youth with type 1 diabetes. This intervention adopted a balanced approach considering both adaptation and fidelity to foster a community of practice to facilitate implementing quality improvement initiatives for improving transition to adult diabetes care. This approach may be adapted for improving transition care for youth with other chronic conditions and to other complex AF-based interventions.

**Trial registration:**

ClinicalTrials.gov NCT03781973. Registered 13 December 2018. Date of enrolment of the first participant to the trial: June 1, 2019.

**Supplementary Information:**

The online version contains supplementary material available at 10.1186/s43058-024-00563-2.

Contributions to the literature

- Multi-site studies of complex quality improvement interventions often find variation in implementation and effects across sites. Better understanding regarding how to balance fidelity and adaptation could advance approaches to successful implementation of complex interventions across multiple sites.
- This paper describes the implementation of an audit and feedback-based intervention which prioritized adaptation while still encouraging fidelity to core components
- Results from this study, which explores the balance between fidelity and adaptation to facilitate engagement, will be important to other investigators developing complex quality improvement interventions.

## Background

Multi-site studies of complex quality improvement (QI) interventions often find variation in implementation and effects across sites. The fit between an intervention and its local context may affect its implementation and effectiveness [1]. Fidelity—the degree to which an intervention is delivered, received, and enacted as intended by its developers—is associated with intervention outcomes [2, 3]. However, adaptation, the “deliberate alteration of (an intervention)’s design or delivery to improve its fit in a given context”, is common under natural conditions [4–10]. At the same time, adaptations may be necessary to increase the relevance and efficacy of a given intervention, fit into local workflows, and facilitate engagement [11].

Fidelity is a multidimensional concept which can be considered at the level of the intervention designer, provider, and recipients [3]. Evaluating the fidelity of an intervention at all levels is essential for understanding its effectiveness. Researchers have stated that both fidelity and adaptation are necessary for high-quality implementation [12–16]. Exploring this balance between fidelity and adaptation in complex multi-faceted and multi-site interventions is needed to understand whether an intervention is employed as intended.

We used an example of a multi-faceted, multi-site intervention to explore the balance between fidelity and adaptation. Bridging the Gap is a quasi-experimental pre-post study with a control group to test the effectiveness of an audit and feedback-based (AF) QI intervention designed to improve diabetes management in the first year after youth transfer from pediatric to adult diabetes care, targeting pediatric healthcare providers at five centers within the Ontario Pediatric Diabetes Network, Canada [17]. Adolescent and young adults (AYA), living with diabetes, face various patient, provider, and health system-related barriers in transitioning to adult-focused care settings [18]. Compared to adults, they are at a high risk of dropping out of medical care, having acute diabetes complications, and of suboptimal glycemic management which confers an increased risk of chronic diabetes complications [19–22].

We aimed to explore how Bridging the Gap study activities exhibited fidelity to the intervention and how they were adapted in a pragmatic real-world setting to understand how intervention components were delivered, received, and enacted, and to account for variation between study sites.

## Methods

### Design

This study was conducted as part of a process evaluation of the Bridging the Gap intervention, a full description of which can be found in its protocol [17]. A forthcoming paper reports on a set of interviews conducted with healthcare practitioners from the five study sites, focusing on their experiences and perspectives during Bridging the Gap. Here, we compare the research team’s planned and actual implementation of the intervention. We also describe the activities undertaken at each study site, detailing if and how they changed throughout the study. We used a previously established approach to examine how study activities exhibited fidelity to the intervention and how they were adapted.

### Setting

Bridging the Gap was conducted at five pediatric diabetes centers (three tertiary and two large community centers) in urban areas in the province of Ontario, Canada. Ontario publicly funds physician services which includes diabetes care. We describe study activities at each site from May 21, 2019, to March 30, 2022.

### Intervention

Bridging the Gap leverages the Got Transition framework, an evidence-driven resource designed to improve the process of transition from pediatric to adult care [23]. Got Transition’s Six Core Elements of Health Care Transition 2.0 is an updated version of the first iteration of this framework and includes the basic components of a structured transition process. These elements are as follows: (1) transition policy; (2) transition tracking and monitoring; (3) transition readiness; (4) transition planning; (5) transfer of care; and (6) transfer completion. Each participating study site was encouraged to pursue its own QI initiative relevant to its context and informed by the Got Transition framework and feedback it received as part of the AF process. In AF, an individual or group’s performance (i.e., the quality of care) is measured, compared to established targets, and performance feedback is given [24]. Coaching and QI resources were provided via webinars and feedback reports. At the start of the intervention, each site completed the Got Transition “Current Assessment of Health Care Transition Activities” to measure their baseline self-assessment of health care transition activities on a scale between 1 (basic) and 4 (comprehensive). After completing the current assessment tool, sites were coached by the research team to develop a “change idea”, an actionable, specific idea for changing a process related to one or more Got Transition elements. Diabetes teams then developed a SMART (Specific, Measurable, Applicable, Realistic, and Timely) aim statement describing their QI initiative’s tangible goals and intended outcomes. Progress on each site’s QI initiative was tracked through site progress reports, wherein providers at participating sites described the progress on their respective initiative and completed the Got Transition Health Care Transition Process Measurement Tool, an objective measure of implementation of the Got Transition elements [24]. The tool includes clear criteria and structured guidelines for scoring each element. The scale for the Transition Process Measurement tool differs from the Current Assessment of Health Care Activities tool—scores for each transition element vary according to complexity or importance (see Supplementary materials). Feedback reports compiled by the research team were provided to each site during the study (Additional file 1). They included data about patient baseline characteristics, transition experience measures such as feelings of transition preparedness and satisfaction with the transition to adult diabetes care, and outcomes such as most recent hemoglobin A1c, a measure of glycemic management. Each site received a report about their patients compared to data about patients at all other study sites combined. The report provided guidance about how to use the data, and there were opportunities to discuss the reports at subsequent webinars. Diabetes teams at study sites were invited to attend seven webinars hosted by the research team, held approximately once every 6 months, over the course of the intervention. Webinars allowed providers to reflect on their study site’s feedback reports and to discuss the challenges and successes of their QI initiatives with diabetes team members at other study sites. Webinars were scheduled on different days of the week and times to coincide with regular diabetes team meetings and to facilitate attendance. Quorum, an online study portal hosted on the Health Quality Ontario website, was used to facilitate sharing of QI resources and to host a private discussion forum for diabetes team members at study sites to ask questions and share information about their progress.

The intention was for the research team to host webinars and facilitate discussion about the feedback reports, to provide coaching about QI initiatives, and to promote sustained engagement with the intervention. Sites were given autonomy over the design and modifications to the QI initiatives as long as they were focused on one or more of the Got Transition core elements. The research team and participating diabetes team members at other sites were available during webinars for consultation and to respond to comments posted on Quorum. We anticipated that sites would focus their QI initiative on one or more Got Transition Core Elements for which their score on the Got Transition Current Assessment of Health Care Transition Activities suggested a need for improvement that was an area of interest to the study site and which could be feasibly addressed. The Bridging the Gap AF intervention was intentionally designed to allow study sites both the structure and flexibility to adapt their specific QI initiative to meet their needs and consider their local context.

### Data collection

The following data sources were used to describe site-specific activities: feedback reports disseminated to each site, QI progress reports, Got Transition measurement reports (Current Assessment of Health Care Transition Activities and Health Care Transition Process Measurement Tool), and webinar summaries [24]. The following data were extracted: narrative descriptions of the study site-specific QI interventions, webinar attendance (the total amount of attendees per session), Got Transition Current Assessment of Health Care Transition Activities scores, Got Transition Health Care Transition Process Measurement Tool scores, and the content of the feedback reports.

### Assessing fidelity and adaptation

We examined how study activities exhibited fidelity to the intervention and how they were adapted. Fidelity was assessed on three different levels as defined by Bellg and colleagues: delivery, receipt, and enactment [3]. We defined fidelity of intervention delivery as whether the research team delivered the intervention to study sites as intended. We defined fidelity of receipt as whether the diabetes team members at participating sites engaged with the intervention. We defined fidelity of enactment as whether participants used feedback provided from the intervention to adjust the way they delivered transition care. Assessing fidelity across these dimensions allowed for a comprehensive examination of adaptations made to the research team-facilitated, AF-based intervention and site-specific QI initiatives.

Fidelity of receipt was assessed by gauging the extent of engagement and interaction with the intervention components. This included webinar attendance and completion of QI progress reports and the Got Transition Health Care Transition Process Measurement Tool. The degree of interaction was further assessed through participation on the Quorum portal, specifically through contributions to the discussion board and the number of files uploaded to shared resources folders.

We used the Framework for Reporting Adaptations and Modifications to Evidence-based Implementation Strategies (FRAME-IS) to assess fidelity of delivery and enactment [25]. Fidelity of both delivery and enactment were assessed in terms of adherence to the intervention’s core elements (i.e., the “key active ingredients”).

Recognizing that modifications can be integral to the successful implementation of an intervention, FRAME-IS is a tool which enables the standardized documentation of adaptations and modifications made to the AF-based interventions, QI supports delivered by the research team (delivery), and the local quality improvement strategies each study site pursued (enactment). FRAME-IS encompasses five domains: (1) adaptation and modification details, (2) implementation strategy, (3) implementation outcomes, (4) context, and (5) reporting. FRAME-IS has been used previously to document an intervention’s development and to elucidate the implementation process [26–28]. FRAME-IS comprises four core modules and three optional modules. We used the modules to guide our understanding of the modifications made to the intervention and to document the details of each modification.

## Results

### Fidelity of delivery

The COVID-19 pandemic temporarily halted the recruitment of the early-implementation cohort, which caused a delay in conducting one of the study webinars as diabetes team members at participating sites had competing priorities. Other than these delays, the intervention was delivered as intended. Seven webinars were held over 3 years, occurring approximately every 6 months, except for a 9-month gap between the final two webinars. Webinar agendas were initially formally planned to discuss the Got Transition core elements and QI methods, with each session focusing on one or two transition elements and a component of QI methodology. These more formal agendas were adapted for the final three webinars, to allow team members at study sites to share their progress on their QI initiatives and discuss how their transition care processes had adapted to the COVID-19 pandemic. Feedback reports were disseminated to study sites 1–2 weeks prior to webinars, beginning with the third webinar. A total of five feedback reports were distributed between November 2019 and March 2022. Quorum, the online study portal operated by Health Quality Ontario, remained optimally functional throughout the study. The study team posted resources on Quorum related to QI methods discussed in the first few webinars and also posted in the chat to encourage others to participate and generate discussion.

### Fidelity of receipt

The extent of engagement and interaction with the intervention varied according to component. There were 22–33 diabetes team members from study sites in attendance at each webinar. At least one representative from each site was present at each webinar. The feedback reports were delivered before each webinar. We did not collect specific information about how each study site engaged with and used the reports. There were 33 posts to the Quorum discussion portal. Of these, 18 were prompts posted by the research team.

### Fidelity of enactment

Each site selected one or more of the Got Transition elements to focus their quality improvement initiative. Table S1 displays the self-assessment score for each Got Transition element from the Current Assessment of Health Care Transition Activities and the element(s) selected by each study site (Additional file 2). Each site completed QI progress reports approximately every 6 months during the intervention. Four progress reports were submitted by each site during the intervention, from May 2020 to December 2021. The data in the site QI reports, highlighting emergent barriers and adaptations to local QI plans, has been summarized elsewhere (Additional file 2: Table S2). Table S3 describes the Got Transition Process Measurement Tool scores for each site once the study was underway and then at the end (Additional file 2). Scores either increased or stayed the same across each element at all sites except one.

Of the five sites, sites 1 and 4 implemented their QI initiatives as planned (Additional file 2: Table S4). For sites 2, 3, and 5, adaptation outcomes were documented using the FRAME-IS framework. Site #2 modified its initiative to add transition tracking flowsheets, orders for transition clinic follow-up appointments, reminders in the electronic medical record to flag patients overdue for a visit, handouts to provide information on transition clinic visits to patients, and patient satisfaction surveys to collect feedback on transition clinic visits. These changes were implemented to optimize the efficiency and flow of transition clinic visits. The original plan for delivery of the content and format of the transition clinics were not altered. The fidelity of these modifications to the planned QI initiative and Got Transition core elements remained consistent. Site #3 expanded its transition policy document after receiving feedback from youth living with T1D attending their clinic. Although the content of the intervention was modified, fidelity to the Got Transition elements of interest remained consistent. Site #5 modified its intervention to target a different Got Transition element, from transition tracking and monitoring to transition planning. The intervention changed from a transition tracking tool to a framework for age-based transition education sessions. At all three study sites that modified their interventions, the study site investigator and diabetes teams participated in the decision to modify the intervention.

## Discussion

We describe implementing a multi-center AF-based intervention across five sites in Ontario to improve the transition from pediatric to adult diabetes care for youth with type 1 diabetes. Based on the local context and shifting priorities, each site followed a unique trajectory in implementing its QI initiatives during the study. We observed that sites took advantage of the study design, allowing for the adaptation of site-specific QI initiatives. Each site designed an initiative suited to its local context based on needs identified using the Got Transition Current Assessment of Health Care Transition Activities and adapted it over time as needs and priorities shifted. The current assessment scores were universally low across all sites and core elements. Therefore, they may not have been an important factor for sites in selecting the core element to focus their QI initiative. For example, site #3 scored lowest on transition readiness and transfer completion but chose to focus their initiative on transition tracking and monitoring; this may have been due to feasibility, the local context, and needs identified by the diabetes team. We observed that most sites converged upon focusing their initiatives on a single transition element. This may have been because focusing on one transition element at a time proved to be the most feasible given the competing responsibilities of diabetes team members. This is exemplified by site #5, which initially focused on a transition tracking and monitoring tool before moving to a different Got Transition element, namely transition planning once an updated electronic medical record system enabled the systematic identification of potentially eligible patients to receive targeted transition readiness education.

The intervention’s core elements were QI initiatives based on the Got Transition framework, webinars, Quorum, and feedback reports. These core elements were not significantly modified over time, except the planned content of the webinars. Webinar agendas were modified to increase participation and respond to how sites had modified their transition care practices in response to the COVID-19 pandemic. Considering modifications to QI initiatives, we distinguish between adaptations made by each site team in response to feedback reports and webinar discussions and adaptations made in response to external factors (i.e., the pandemic-related changes to transition care practice). The study was launched in May 2019; however, the onset of the COVID-19 pandemic in March 2020 caused significant delays to the rollout of some initiatives due to shifting priorities and staffing shortages as well as the recruitment of the early implementation cohort. However, the AF-based intervention was implemented as originally planned, and fidelity of delivery was preserved.

Fidelity of enactment was preserved during the study. Based on feedback, sites #2 and 3 introduced minor modifications to their interventions. Only one site, modified its intervention to focus on a different Got Transition core element. Study sites were permitted to adapt their intervention; therefore, fidelity of enactment was preserved. Attendance at webinar sessions was variable, although at least one member from each participating site attended every webinar session and received feedback from site investigators, demonstrating fidelity of receipt. There were no a priori expectations of webinar attendance, although at least one representative from each site was encouraged to attend each webinar. Each study site, except one, had an improved score on the process measurement assessments in at least one core Got Transition domain compared to the initial score, demonstrating improvement in self-assessment of transition process measures. Given that the intervention was implemented over 2 years, it is likely that there were different diabetes team members completing the self-assessment tools over time. There could have been inconsistencies in the interpretation of scores between individuals or it is possible that transition processes changed over time in a negative direction, offering a potential explanation as to why this score decreased.

Through interaction with study components, namely webinars and feedback reports wherein participants were encouraged to share knowledge and experiences, our study facilitated a community of practice (CoP), supporting the implementation of the AF-based intervention through fostering engagement and thus preserving fidelity of enactment. CoPs improve organizational performance, promote the dissemination of knowledge, and facilitate the uptake of EBPs within healthcare [29–37]. CoPs are characterized by their dynamic and informal nature, possessing a flexible and fluid membership as opposed to a defined group of participants and addressing challenges as they arise to create a direct link between learning and performance [29, 30]. Participating sites engaged with the Bridging the Gap intervention to improve the transition to adult care. Webinars were held bi-annually, allowing participants to discuss their progress, challenges, and successes. Finally, feedback reports enabled participants to reflect upon their interventions and modify them, if needed. CoPs require commitment and enthusiasm and are sustained through shared interests. Organizers should foster CoPs by not imposing a specific structure or membership requirement [38–40]. CoPs provide a useful avenue for promoting engagement among members. However, the outputs of CoPs are variable due to the bottom-up nature of their structure [41]. Based on webinar attendance and the execution of QI initiatives, there was evidence of a high level of engagement. Participants were able to use the webinars to learn about each other’s QI initiatives as well as challenges which they faced in transition care. The sharing of common experiences and discussions about the QI initiatives during webinars and on Quorum facilitated the emergence of a community of practice founded upon a shared repertoire of frameworks, tools, and languages to accomplish collective goals through joint discussion.

Both adaptation and fidelity are necessary to successfully implement an intervention [12–16]. There has been much written about the importance of fidelity to an intervention, however, less about the notion of adaptation. This process evaluation contributes to an area within implementation science which seeks to advance a science of adaptation, recognizing the importance of “ongoing adaptation of interventions during implementation due to dynamic settings and needs” [41]. Thus far, adaptation and fidelity have been viewed as two opposing concepts requiring separate study. However, our study seeks to analyze these processes in tandem, positing that both are required for the successful implementation of an intervention. As noted by David Chambers, there is a need for health interventions which are designed with an empirically supported “immutable” core as well as an “adaptable periphery”, encouraging a proactive approach to adaptation and thus enabling their equitable implementation [42]. The BTG study balanced adaptation and fidelity by planning for and providing sites with the flexibility for adaptation, allowing them to pursue QI initiatives that they deemed important, thus enabling each site to remain engaged and preserving fidelity of receipt and enactment. Allowing choice within a clearly defined, core set of transition elements gave structure to the development of the QI initiatives, preserving fidelity of delivery.

Our exploration of fidelity and adaptation has some limitations. Information was collected retrospectively from meeting minutes, webinar summaries, QI progress reports, and feedback reports, making it challenging to discern the motivations for modifications made to QI initiatives. Minutes analyzed retrospectively may not have captured all the relevant information discussed at a given meeting. We do not know how sustainable the QI interventions initiated as part of the Bridging the Gap study will be over time. As Laur and colleagues have discussed, the successful implementation of an intervention involves a “balancing act” between following rigorous research methods while also adapting to a specific, changing context [43]. Furthermore, these interventions should be grounded in theory, involve relevant stakeholders, and consider equity. While our study included all these components to ensure the successful implementation of QI initiatives, we did not include a focus on sustainability in our coaching to study sites. We also did not provide them with external financial resources to ensure the implementation of initiatives. Future studies should seek to assess the long-term feasibility of such programs, incorporating discussions on sustainability where applicable.

We used the FRAME-IS framework to characterize dynamic, provider-led modifications to the BTG intervention within a CoP. FRAME-IS is particularly useful because its application identifies and assesses site-specific adaptations. The framework has been validated through its use in other studies seeking to examine modifications in implementation strategies [26–28]. However, we were limited in using FRAME-IS because we had a finite amount of data which was analyzed retrospectively to guide our documentation of adaptations made during the study. The challenges we faced in applying this framework retrospectively have been noted by others who have used it. Zehner and colleagues state that FRAME-IS was useful in retrospectively identifying several planned, system-wide adaptations but may not have captured as many local, unplanned modifications [26]. While we were ultimately able to characterize adaptations using the FRAME-IS modules, it was challenging to categorize certain modifications such as with site #5, which shifted to a new QI initiative entirely to focus on a new Got Transition element of interest. While the initiative itself was modified, fidelity to the BTG study protocol was preserved. Future studies should plan to document implementation outcomes of similar interventions in real-time using checklists modeled on the FRAME-IS modules, enabling a deeper understanding of the rationale for adaptations made iteratively as a given intervention is being implemented. Despite these limitations, our results provide important insights into how adaptation and fidelity can be balanced successfully in complex QI interventions.

## Conclusions

We describe implementing a multi-center AF-based intervention across five sites in Ontario to improve the transition from pediatric to adult diabetes care for youth with type 1 diabetes. This intervention adopted a balanced approach considering both adaptation and fidelity to foster a community of practice to facilitate implementing quality improvement initiatives for improving transition to adult diabetes care. This approach may be adapted for improving transition care for youth with other chronic conditions and to other complex AF-based interventions.

### Supplementary Information

**Additional file 1.** Feedback Report Template.

**Additional file 2: Table S1.** Got Transition Current Assessment of Health Care Transition Activities Baseline Element Scores and Chosen Elements Targeted by Interventions. **Table S2.** Site Quality Improvement Initiative Summaries. **Table S3.** Transition Performance of Each Site Before and After Health Care Transition Process Measurement Tool Implementation. **Table S4.** Adaptations and modifications to site QI initiatives documented using the FRAME-IS framework.

## Data Availability

All data generated or analyzed during this study are included in this published article (and its supplementary information files).

## Abbreviations

QI: Quality improvement
AF: Audit-and-feedback
FRAME-IS: Framework for Reporting Adaptations and Modifications to Evidence-based Implementation Strategies
CoP: Community of practice
