# Supplementary material for: An assessment of adaptation and fidelity in the implementation of an audit and feedback-based intervention to improve transition to adult type 1 diabetes care in Ontario, Canada
Source: Implement Sci Commun. 2024 Mar 18;5:25. doi: 10.1186/s43058-024-00563-2 (PMC10946155; doi:10.1186/s43058-024-00563-2)
Supplement: Supplementary file 2 — Additional file 2: Table S1. Got Transition Current Assessment of Health Care Transition Activities Baseline Element Scores and Chosen Elements Targeted by Interventions. Table S2. Site Quality Improvement Initiative Summaries. Table S3. Transition Performance of Each Site Before and After Health Care Transition Process Measurement Tool Implementation. Table S4. Adaptations and modifications to site QI initiatives documented using the FRAME-IS framework. [file 43058_2024_563_MOESM2_ESM.docx]

**Tables and Figures**

**Table S1. Got Transition Current Assessment of Health Care Transition Activities Baseline Element Scores and Chosen Elements Targeted by Interventions**

|  | **Got Transition Element Baseline Scores (Out of 4)** | | | | | | **Chosen Got Transition Elements Targeted by Interventions** |
| --- | --- | --- | --- | --- | --- | --- | --- |
| **Sites** | **Transition Policy** | **Transition Tracking and Monitoring** | **Transition Readiness** | **Transition Planning** | **Transfer of Care** | **Transfer Completion** |  |
| Site #1 | 2 | 2 | 2 | 2 | 2 | 2 | Transition Readiness |
| Site #2 | 2 | 2.5 | 2.5 | 2.5 | 2.5 | 2.5 | Transition Tracking and Monitoring, Transition Readiness, Planning, and Transfer of Care |
| Site #3 | 2 | 2 | 1 | 2 | 2 | 1 | Transition Tracking and Monitoring |
| Site #4 | 2 | 3 | 2 | 2 | 2 | 2 | Transition Tracking and Monitoring |
| Site #5 | 1 | 1 | 2 | 2 | 2 | 1 | Transition Tracking and Monitoring, Transition Planning |

**Table S2. Site Quality Improvement Initiative Summaries**

| Site | Intervention | Description | Progress |
| --- | --- | --- | --- |
| Site #1 | Transition Readiness Questionnaire, tracking tool | To track patients ages 14-17 to target them for transition readiness education, distributing Transition Readiness Questionnaire | Between January 7, 2020 and March 3, 2020 they reached 110 patients -- a 77% response rate. The implementation of the tracking tool and surveys was halted due to COVID-19 pandemic until November 2020, and again in December 2020. Initiative halted until the end of the study. |
| Site #2 | Transition (T-STAR) Clinic, tracking tool | Dedicated transition self-management, teaching and review clinic for all patients with T1D aged 15.5-18 once per year, tracking tool | COVID-19 pandemic delayed implementation of clinic until November 2020. Changes such as creation of transition flowsheet, implementation of follow-up order in electronic medical record, and patient feedback surveys (more detail in Table 4) introduced to clinic over the duration of the study to make visits more effective. |
| Site #3 | Transition-Q Questionnaire | Tracking tool | Initiative halted in the wake of COVID-19 pandemic as team shifted focus to developing streamlined process for booking virtual appointments. QI initiative not resumed as of end of study. |
| Site #4 | Transition Policy Document, tracking tool | Implementation of tracking tool, distribution of transition policy document providing patients with list of adult care providers’ contact information to reduce amount of patients over age of 19 years old | Document revised and updated to reflect targeted focus areas after feedback. Staffing changes in second half of 2021 brought initiative to a halt. |
| Site #5 | "Ready, Set, Go!" Framework | Framework for milestone-based future transition education sessions with developmentally appropriate objectives: Ready (grades 8-9), Set (grades 10-11), Go (grades 11-12) | Originally focused on transition tracking and monitoring, then adjusted QI activities to focus on transition planning. Due to staff shortages and pandemic restrictions, initiative was delayed |

**Table S3:** **Transition Performance of Each Site Before and After Health Care Transition Process Measurement Tool Implementation**

|  | **Transition Policy (n/14)** | | **Transition Tracking and Monitoring (n/7)** | | **Transition Readiness (n/7)** | | **Transition Planning (n/14)** | | **Transfer of Care (n/6)** | | **Transfer Completion (n/3)** | |
| --- | --- | --- | --- | --- | --- | --- | --- | --- | --- | --- | --- | --- |
| **Site** | **Initial** | **Final** | **Initial** | **Final** | **Initial** | **Final** | **Initial** | **Final** | **Initial** | **Final** | **Initial** | **Final** |
| Site #1 | 1 | 6 | 6 | 7 | 7 | 7 | 6 | 4 | 2 | 5 | 0 | 0 |
| Site #2 | 0 | 8 | 5.5 | 7 | 0 | 7 | 2 | 6 | 1.5 | 2 | 0 | 3 |
| Site #3 | 0 | 4 | 0 | 3 | 0 | 6 | 6 | 9 | 2 | 4 | 0 | 2 |
| Site #4 | 4 | 10 | 7 | 7 | 0 | 0 | 8 | 8 | 2 | 2 | 0 | 0 |
| Site #5 | 0 | 0 | 1 | 0 | 0 | 0 | 1 | 9 | 0 | 2 | 0 | 0 |

**Table S4: Adaptations and modifications to site QI initiatives documented using the FRAME-IS framework**

| **Sites** | **Briefly describe the evidence-based practice, implementation strategy, and modification** | **What is modified?** | **Nature of the modification / Relationship to core elements** | **Goal / level of the modification** | **When is it initiated? Planned?** | **Who participated in the decision to modify?** | **How widespread is modification?** |
| --- | --- | --- | --- | --- | --- | --- | --- |
| Site #2 | EBP: Transition readiness, planning, and transfer of care.    Implementation strategy being modified: Transition Self-Management, Teaching and Review clinic for all patients with T1D aged 15.5-18.    Modifications being made are: introduction of transition tracking flowsheet with list of ideas to be discussed, patient feedback surveys, order for TSTAR follow-up, reminders in electronic medical record, Smartphrase in electronic medical record. | Content: Changed (details in Module 3).  Evaluation: unchanged.  Training: unchanged.  Context: unchanged. | Adding elements: addition of content in the form of tracking list with ideas to be discussed, patient feedback surveys.    Fidelity to core elements consistent; the core Got Transition goals were still being targeted and the main intervention remained the same; changes implemented served to ameliorate the process. | Goal: To increase the clinical effectiveness of the EBP, allowing improvement of TSTAR clinics.    Level: organizational level, clinician level | Implementation phase | Researchers | Clinic/unit |
| Site #3 | EBP: Transition tracking and monitoring.    Implementation strategy being modified:  transition policy document providing patients with list of adult care providers’ contact information to reduce amount of patients over age of 19 years old.    Modifications being made: content changed to reflect targeted focus areas after feedback. | Content: changed.  Evaluation: unchanged.  Training: unchanged.  Context: unchanged. | Tailoring/tweaking/refining.    Fidelity to core elements consistent; the core Got Transition goals were still being targeted and the main intervention remained the same; changes implemented served to ameliorate the process. | Goal: To increase the acceptability, appropriateness, or feasibility of the implementation effort.    Level: patient or other recipient level. | Implementation phase | Researchers | Group of patients or other recipients for whom the EBP is being implemented |
| Site #5 | EBP: Transition tracking and monitoring, changed to transition planning.    Implementation strategy being modified: transition tracking tool changed to framework for milestone-based future transition education sessions with developmentally appropriate objectives: Ready (grades 8-9), Set (grades 10-11), Go (grades 11-12).    Modifications being made: entire intervention shifted to implement a new EBP. | Content: changed. Context: format changed. | Drift from the implementation strategy followed by a return to strategy within the implementation encounter. Fidelity: consistent; Switched to different Got Transition element. | Goal: Other; To reflect changed Got Transition element of focus | Implementation phase | Researchers | Clinic/unit, group of patients or other recipients that share a particular characteristic. |

EBP = Evidence-based practice
